# Recently discovered *Aedes japonicus japonicus* (Diptera: Culicidae) populations in The Netherlands and northern Germany resulted from a new introduction event and from a split from an existing population

**DOI:** 10.1186/s13071-015-0648-1

**Published:** 2015-01-22

**Authors:** Dorothee E Zielke, Adolfo Ibáñez-Justicia, Katja Kalan, Enrih Merdić, Helge Kampen, Doreen Werner

**Affiliations:** Institute for Land Use Systems, Leibniz-Centre for Agricultural Landscape Research, Eberswalder Straße 84, 15374 Muencheberg, Germany; National Centre for Monitoring of Vectors, Netherlands Food and Consumer Product Safety Authority, Ministry of Economic Affairs Wageningen, Wageningen, The Netherlands; University of Primorska, Koper, Slovenia; University of Osijek, Osijek, Croatia; Friedrich-Loeffler-Institut, Federal Research Institute for Animal Health, Insel Riems, Greifswald, Germany

**Keywords:** *Aedes japonicus japonicus*, Asian bush mosquito, Europe, Microsatellites, Population genetics, *nad*4 haplotypes

## Abstract

**Background:**

Originally native to East Asia, *Aedes japonicus japonicus*, a potential vector of several arboviruses, has become one of the most invasive mosquito species in the world. After having established in the USA, it is now spreading in Europe, with new populations emerging. In contrast to the USA, the introduction pathways and modes of dispersal in Europe are largely obscure.

**Methods:**

To find out if two recently detected populations of *Ae. j. japonicus* in The Netherlands and northern Germany go back to new importations or to movements within Europe, the genetic makeup of mosquito specimens from all known European populations was compared. For this purpose, seven microsatellite loci from a representative number of mosquito specimens were genotyped and part of their mitochondrial *nad*4 gene sequenced.

**Results:**

A novel *nad*4 haplotype found in the newly discovered Dutch population of *Ae. j. japonicus* suggests that this population is not closely related to the other European populations but has emanated from a further introduction event. With five *nad*4 haplotypes, the Dutch population also shows a very high genetic diversity indicating that either the founder population was very large or multiple introductions took place. By contrast, the recently detected North German population could be clearly assigned to one of the two previously determined European *Ae. j. japonicus* microsatellite genotypes and shows *nad*4 haplotypes that are known from West Germany.

**Conclusion:**

As the European populations of *Ae. j. japonicus* are geographically separated but genetically mixed, their establishment must be attributed to passive transportation. In addition to intercontinental shipment, it can be assumed that human activities are also responsible for medium- and short-distance overland spread. A better understanding of the processes underlying the introduction and spread of this invasive species will help to increase public awareness of the human-mediated displacement of mosquitoes and to find strategies to avoid it.

## Background

The Asian bush mosquito *Aedes* (*Finlaya*) *japonicus japonicus* (Theobald, 1901) (*Hulecoeteomyia japonica japonica* sensu Reinert *et al*. [1]) is one of the most expansive mosquito species in the world [2]. After repeated interceptions in New Zealand in the early 1990s [3], the first established populations outside the original distribution range were detected in the eastern USA [4,5]. From three states that had initially been invaded, it spread in only a few years into 30 further states, including Hawaii [6-8]. Today, the species is also present in Canada [9].

In Europe, larvae of *Ae. j. japonicus* were first detected in the year 2000 in a used tyre trade company in France, but were eradicated [10]. In 2002, the mosquito was found in Belgium, again in the context of a used tyre platform. The species was still present in 2003 and 2004 and was found during a national mosquito monitoring programme in 2007 and 2008, when a second company trading with used tyres was affected in the same Belgian town [11]. As *Ae. j. japonicus* was never caught more than 2 km away from these two locations, it was concluded that the population was not expanding.

Also in 2008, larvae of *Ae. j. japonicus* were discovered in northern Switzerland and in several places on the German side of the Swiss-German border [12]. A monitoring programme carried out in 2009 and 2010 in the German federal state of Baden-Wurttemberg showed that the Asian bush mosquito had already infested a large area along the border with Switzerland [13]. Another study from 2010 detected its presence near the city of Stuttgart, approximately 80 km north of what had been assumed to be the northern distribution limit of the species [14]. Several findings of *Ae. j. japonicus* individuals made in 2011 at various places in Baden-Wurttemberg (Werner & Kampen, unpublished) indicated a much greater distribution area at that time. In the same year, a population was found widely distributed on both sides of the Austrian-Slovenian border [15]. By 2013, this population had expanded over the entire country of Slovenia, even reaching northern Croatia (Kalan, Merdić, unpublished).

Since its first detection in Baden-Wurttemberg in 2008, *Ae. j. japonicus* seems to have been spreading continuously across Germany. Populations have been shown to exist in the federal states of North Rhine-Westphalia and Rhineland-Palatinate [16] and, more recently, as far north as in Lower Saxony [17]. In January 2013, Dutch researchers detected a single *Ae. j. japonicus* female already trapped in July 2012 during routine monitoring in the municipality of Lelystad (province of Flevoland). Extensive surveillance from April to October 2013 in the surroundings of the first finding identified numerous breeding sites over large parts of the municipality [18]. The first finding was about 7 km from a tyre trading company, but no individuals could be found on the company’s premises.

Of the six *Ae. j. japonicus* populations detected in Europe, only the Belgian one is known to be due to an importation in used tyres. For the other five populations there is no information on their mode of introduction nor on their origin or relatedness.

*Aedes j. japonicus* is a potential vector of several arboviruses including West Nile virus (WNV) and Japanese encephalitis virus [19,20]. In the USA, it has been found infected with WNV in the field [21]. In addition, the species is able to transmit La Crosse encephalitis, St. Louis encephalitis, eastern equine encephalitis and Rift Valley fever viruses under laboratory conditions [22-24] and is susceptible to infection with chikungunya and dengue viruses [25]. Its spread and behaviour therefore merit close observation.

The objective of the present study was (i) to learn more about the relationships between the various European populations of *Ae. j. japonicus* and, in particular, (ii) to assign the newly discovered populations in Lelystad, The Netherlands, and in Lower Saxony, Germany, to already known genotypes, in order to detect genetic proofs for introduction or migration events. For this purpose we analysed highly polymorphic simple sequence repeats (microsatellites) and maternally inherited, rapidly evolving mitochondrial *nad*4 sequences which are characterised by variable numbers of repeats or nucleotide sequences, making them appropriate targets for population genetics and the identification of source populations [26,27].

## Methods

### Mosquitoes

Mosquito larvae and eggs were collected between May and October 2013 from flower vases and other small artificial water containers and from ovitraps in cemeteries and gardens [28].

**Figure 1:**
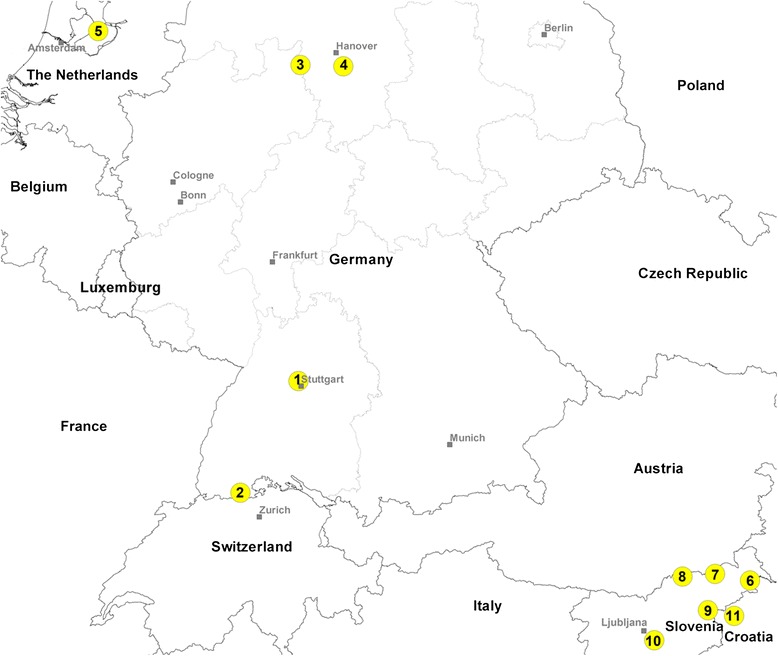
**Geographic distribution of** ***Ae. j. japonicus*** **sampling locations (yellow dots).** Numbers of locations refer to Table 1.

Individuals central to the study were collected from two sites, about 60 km apart, in the North German federal state of Lower Saxony (NG) and from six sites within the municipality of Lelystad, The Netherlands. In addition, specimens from two towns in the South German federal state of Baden-Wurttemberg (SG) and from 18 sites in Slovenia as well as two individuals from two close collection sites in Croatia were examined (for details see Table 1 and Figure 1). For comparison, previously analysed specimens from West Germany (WG), Belgium and Switzerland [29] were included.

**Table 1:** **Origin of** ***Ae. j. japonicus*** **specimens included in the study**

**Country**	**Federal state/province/region**	**Location**	**No. of mosquitoes analysed**		**I**
			**Microsatellites**	***nad*** **4**	
Germany	Baden-Wurttemberg (SG)	Korntal (1)	39	22	1.26
		Waldshut-Tiengen (2)	41	26	1.21
	Lower Saxony (NG)	Bad Eilsen (3)	20	20	1.1
		Sarstedt (4)	12	10	1.15
The Netherlands	Flevoland	Lelystad (5)	43	37	1.06
Slovenia	Pomurska(6)	Ljutomer	5	2	0.67
	Podravska (7)	Selnica ob Dravi	5	4	1.16
		Pesnica pri Mariboru	2	0	
		Sentilj	5	2	
		Lovrenc na Dravskem Polju	2	1	
		Ptuj	3	2	
		Makole	5	5	
		Ormoz	2	2	
	Koroska (8)	Lovrenc na Pohorju	5	4	1.12
		Ribnica na Pohorju	2	2	
		Muta	3	3	
		Slovenj Gradec	5	4	
	Savinjska (9)	Rogatec	2	1	1.23
		Smarje pri Jelsah	2	0	
		Bistrica ob Sotli	5	4	
	Osrednjeslovenska (10)	Ig	3	1	0.74
		Grosuplje	2	2	
		Ivancna Gorica	1	0	
Croatia	Krapina-Zagorje (11)	Djurmanec	1	0	n. a.
		Macelj	0	0	

Out of a total of 215 individuals subjected to microsatellite analysis, 154 produced analysable *nad*4 sequence data. Numbers in parentheses refer to the geographic location of the collection sites as shown in Figure 1. NG = North Germany, SG = South Germany, I = Shannon’s information index, n. a. = not applicable.

Larvae from NG and SG were sampled in cemeteries, taken to the laboratory in their original water and reared to adults. These were killed by exposing them to -20°C for at least 1 h, identified morphologically to species level according to the key by Schaffner *et al*. [30] and kept frozen at -20°C until molecular examination. Dutch individuals were collected from rain water barrels and buckets in allotment gardens and from a flower vase in a cemetery. They were placed as larvae into 80% ethanol immediately after collection in the field and were also identified using the key by Schaffner *et al.* [30]. Slovenian specimens were also collected as larvae from cemeteries and gardens and preserved in 80% ethanol. Identification was made using the key in the ECDC’s guidelines for the surveillance of invasive mosquitoes in Europe [28]. The specimens from Croatia were reared to adults from eggs found in cemeteries and were determined to species using the key by Gutsevich *et al.* [31].

### DNA extraction

DNA extraction was performed on complete adult mosquitoes or larvae using the QIAamp DNA Mini Kit (Qiagen) according to the manufacturer’s instructions. In the case of the Croatian individuals, DNA was extracted from single legs using the same kit. DNA was eluted in 80 μl EB buffer (Qiagen) and kept frozen until use.

### Microsatellite analysis

PCR amplification was performed in a C1000™ 96 well thermal cycler (BioRad). The thermoprofile consisted of a 3 min denaturation step at 94°C, followed by 30 cycles of 30 s at 94°C, 30 s at 56°C and 30 s at 72°C, and a final 10 min elongation step at 72°C. For each of the seven targeted microsatellite loci (OJ5, OJ10, OJ70, OJ85, OJ100, OJ187 and OJ338) one pair of primers was used as previously described [32]. Only the forward primer for locus OJ5 was redesigned [33]. PCR products were sized in a 3130xl Genetic Analyzer (Applied Biosystems/Hitachi), and the obtained fragment length analysis data were visualised and verified with GeneMapper 3.7 (Applied Biosystems).

Because frequency-based microsatellite analysis requires a minimum population size that was not given for every sampling site in Slovenia, individuals from there were assigned to five groups according to the geographic regions where they were sampled.

### *nad*4 sequencing

Additionally, part of the sodium dehydrogenase subunit 4 (*nad*4) gene of the mitochondrial DNA of the sampled specimens was sequenced using a modification of the protocol of Fonseca et al. [32]. The primers used, ND4F (5′-CGTAGGAGGAGCAGCTATATT-3′) and ND4R1X (5′-TGATTGCCTAAGGCTCATGT-3′) [33], amplify a 424 bp fragment between positions 8398 and 8821 in the *Anopheles gambiae* genomic sequence (GenBank accession no. L20934). DNA amplification was preceded by a 10 min denaturation step at 96°C and consisted of 35 cycles of 40 s at 94°C, 40 s at 56°C and 60 s at 72°C. A final extension step of 7 min at 72°C was added. PCR products were checked by electrophoresis on a 1.5% agarose gel run for one hour and visualised by ethidium-bromide staining. DNA bands were excised and recovered with the QIAamp Gel Extraction Kit (Qiagen). Afterwards, they were cycle-sequenced in both directions with the BigDye Terminator v1.1 Cycle Sequencing Kit (Life Technologies). PCR products were cleaned with SigmaSpin Sequencing Reaction Clean-Up Columns (Sigma-Aldrich) before being run on a 3130xl Genetic Analyzer. FASTA files of the obtained sequences were aligned with MultAlin [34] to detect nucleotide polymorphisms.

### Statistical analysis

Microsatellite signatures were subjected to Bayesian cluster analysis of multilocus microsatellite genotypes implemented in the software STRUCTURE 2.0 [35]. Following the method of Evanno [36], the optimal number of clusters was determined using the web-based software STRUCTURE HARVESTER [37].

Nei’s genetic distance and pairwise population *F*_ST_ values were calculated with GenAlEx to perform a principal coordinate analysis (PCoA) [38]. Furthermore, departures from the Hardy-Weinberg equilibrium were examined. Shannon’s information index (I), the mean number of alleles and the observed and unbiased expected heterozygosity were identified using GenAlEx [39]. Shannon’s information index which is used to calculate genetic diversity is a quantity that shows the frequency of each allele in addition to the total number of alleles [39,40]. The higher the Shannon’s information index, the higher the population diversity. As the Shannon’s information index is frequency-based, it is not applicable to single individuals.

## Results

Microsatellites of a total of 215 *Ae. j. japonicus* specimens from the Dutch, German and Slovenian/Croatian populations were analysed. In some cases, previously obtained data from West German, Swiss and Belgian samples [29] were included in the analyses for comparative purposes.

**Figure 2:**
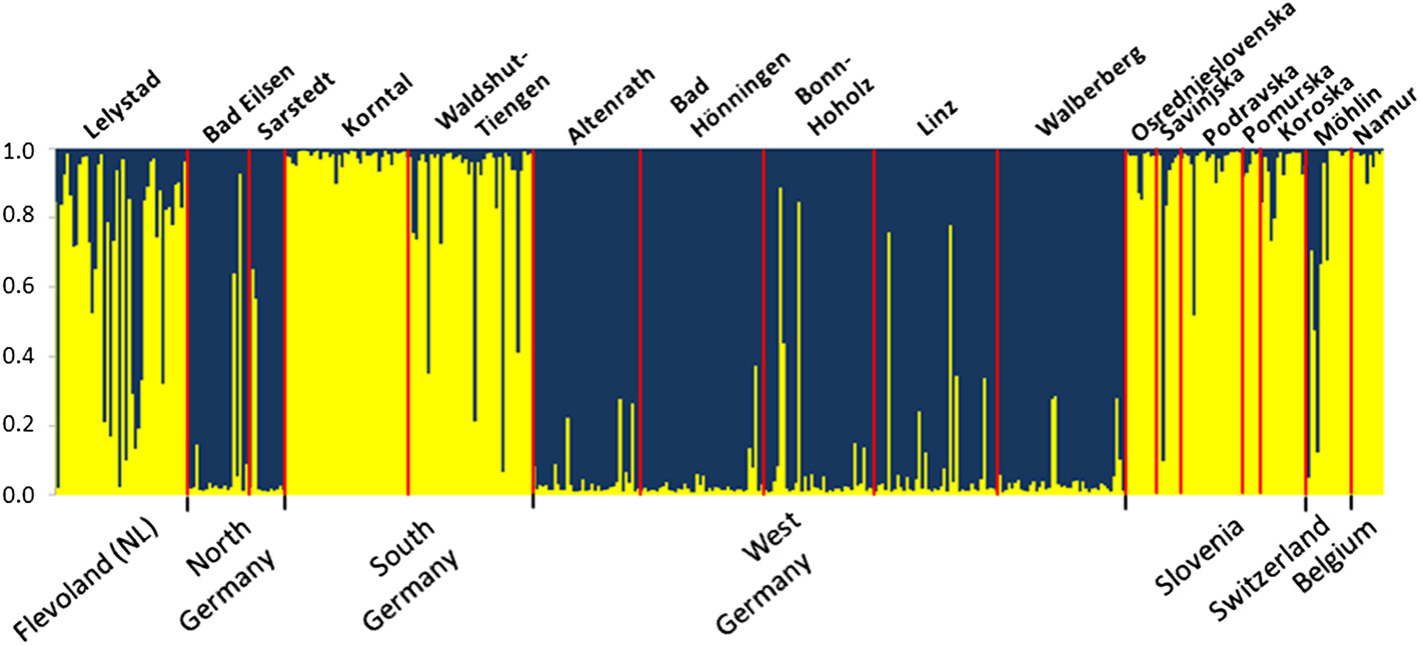
**Results of Bayesian cluster analysis showing the two** ***Ae. j. japonicus*** **genotypes in Europe (yellow = genotype 1, blue = genotype 2).** As the Slovenian and Croatian samples are considered to belong to one and the same population, the Croatian specimen has been added to the samples from Podravska (Slovenia) for the purpose of this presentation. WG, Swiss and Belgian individuals had previously been analysed [29] but are included for comparison.

The obtained microsatellite signatures enabled the examined specimens to be assigned to one of two genotypes described for Germany (Figure 2). The NG population displayed exactly the same genotype 2 as the individuals from WG that had been previously analysed [29]. All other European populations were to be assigned to genotype 1, although the Dutch population showed clear signs of admixture. Only one of the two Croatian individuals available provided analysable microsatellite data. For the statistical analyses, this individual was added to the geographically closest Slovenian region of Podravska. It showed a probability of more than 95% to belong to genotype 1.

A Hardy-Weinberg equilibrium check showed significant deviations at most microsatellite loci of specimens from the two SG sites, Korntal and Waldshut-Tiengen. A few more departures from the Hardy-Weinberg equilibrium, mostly at loci OJ5, OJ10 and OJ187, were found across all populations. A closer inspection of these did not produce considerable effects of null alleles [41]. Deviations were due both to higher than expected and to lower than expected heterozygosity.

**Figure 3:**
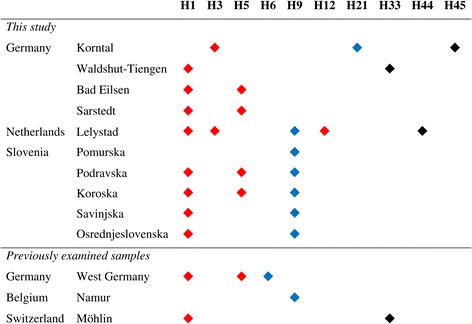
***nad*** **4 haplotypes found at the different sampling locations.** Croatian individuals were excluded due to heteroplasmy. WG, Swiss and Belgian individuals had previously been analysed [29] but are included for comparison. Black = haplotypes described from Europe only, blue = haplotypes also described from the USA, red = haplotypes also described from the USA and Japan.

Of the 215 genotyped individuals, 154 examined for their *nad*4 gene sequence provided analysable results. Nine different *nad*4 haplotypes were obtained: H1, H3, H5, H9, H12, H21, H33, and two haplotypes that have not previously been described (Figure 3). As there are currently 43 *nad*4 haplotypes known for *Ae. j. japonicus* (Fonseca, pers. comm.), we suggest naming the newly found haplotypes H44 and H45. Haplotype sequences are available in [27] and GenBank (accession nos. KJ958405, DQ470159, KM610232 and KM610233, with the latter two being the new ones). H21 and H45 occurred exclusively in Korntal (SG) near the city of Stuttgart in the federal state of Baden-Wurttemberg, while the newly discovered NG population only showed haplotypes H1 and H5. In the Dutch municipality of Lelystad, the most frequent haplotype was H12, which is unique in Europe. Displaying four further haplotypes, the Dutch population was the most heterogeneous one. This observation underlines the admixed microsatellite signature of this population. Among the haplotypes found in The Netherlands, H3 (a single individual) was shared with the Korntal (SG) samples, while H9 (five individuals) was a common haplotype in Slovenia. Surprisingly, the samples from the two SG locations did not share any *nad*4 haplotype, and their microsatellite signatures were different too. The Croatian mosquitoes could not be assigned to a defined haplotype as both specimens were characterised by mitochondrial heteroplasmy, the coexistence of multiple mitochondrial haplotypes in a single organism [42] which is a common phenomenon in *Ae. j. japonicus*.

**Figure 4:**
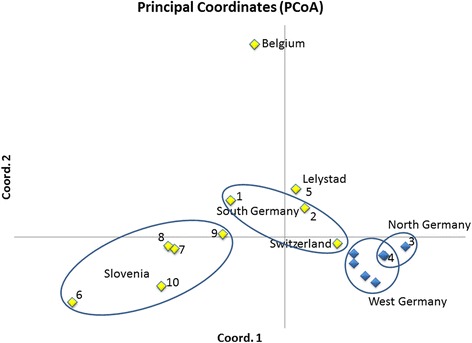
**Principal coordinate analysis plot of pairwise population** ***F***_**ST**_ **values for the European** ***Ae. j. japonicus*** **populations.** Previously analysed samples from West Germany, Switzerland and Belgium [29] are included in this plot to clarify the relationships between all European populations. Locations examined in this study are numbered as in Table 1. Yellow diamonds = genotype 1, blue diamonds = genotype 2. Samples from the same geographically distinct populations are encircled in blue. The diamond representing location 4, which belongs to the NG population, covers another diamond belonging to the WG population.

A principal coordinate analysis based on pairwise population *F*_ST_ values (Figure 4) suggests that the NG and WG samples are very closely related, but stand separate from the SG/Switzerland population. Although belonging to the same genotype 1, samples from the other populations were admixed and displayed a greater genetic distance. Interestingly, the Dutch samples seem to be closely related to those from Waldshut-Tiengen (SG). These, in turn, are more closely related to the previously examined and geographically closest Swiss individuals than to those from Korntal (SG) which are most similar to the Slovenian/Croatian ones. The previously analysed Belgian population [29] stands far apart from all other populations, underlining its geographic and genetic isolation.

## Discussion

A previous study focussing on the West German *Ae. j. japonicus* population established the existence of two distinct but mixed genetic strains of this species in Europe [29]. During that study, mosquito samples from the most recently discovered geographic populations in The Netherlands and northern Germany were not yet available. In addition, only a few specimens from the Slovenian population were included. In the present study, representative numbers of all European populations not previously analysed were considered and, based on Bayesian cluster analysis of their microsatellite data, could be clearly assigned to one of the two genetic strains previously identified in Europe.

The results of Zielke et al. [29] led to the conclusion that the Asian bush mosquito was introduced into Europe on at least two occasions. Samples from the Belgian population [11] show a genotype 1 genetic signature, equal to most of the individuals from the SG and Slovenian/Croatian populations. By contrast, the population detected in western Germany in 2012 appears to be the result of a second introduction of *Ae. j. japonicus* into Europe [29]. Due to microsatellite signatures identical to those of samples from WG, the NG population must be supposed to be an offshoot of the WG population. In summary, the microsatellite analysis (Figures 2 and 4) mirrors the geographic separation of the various European populations.

The mitochondrial haplotypes H1 and H5 found in North Germany are also common in the previously described WG population and support a close genetic relationship. H1 is a common haplotype which can be found in most *Ae. j. japonicus* populations all over the world. Haplotype H5 is also common on the northern Japanese island of Hokkaido [27] but was very rarely found in the USA, suggesting that genotype 2 German *Ae. j. japonicus* might have been introduced from Japan. As adults or larvae, individuals could then have been transported by vehicles along the motorways between the German federal states. This assumption is supported by the fact that the initial findings of *Ae. j. japonicus* in northern Germany were concentrated in cemeteries of towns close to a motorway running northwards from West Germany [17]. An active migration of the species from West to North Germany can be excluded because the two populations are geographically separated.

Samples from South Germany differ significantly from each other in their genetic makeup although forming one geographic population. Individuals from Waldshut-Tiengen (SG) are apparently more closely related to the Swiss samples of the same geographic population than to the Korntal (SG) samples, as only the first two of them show haplotype H33. This haplotype has not been reported from the USA, and in Europe has so far only been found in Swiss specimens [29]. This suggests an introduction of at least a few individuals from Asia. By contrast, individuals from Korntal (SG) are characterised by haplotypes H3, H21 and H45. The latter two haplotypes have been found exclusively in the Korntal (SG) samples. This might indicate a separate introduction of mosquitoes into this area. H3 was first detected in individuals from the southern Japanese islands of Honshu and Kyushu [27] whereas H21 was previously found in some Pennsylvanian individuals [32]. Furthermore, the Korntal (SG) samples show a very clear microsatellite signature of genotype 1 with almost no admixture of genotype 2 (Figure 2).

To date, Lelystad in The Netherlands is considered to be the northernmost location infested by *Ae. j. japonicus* in Europe. The Dutch population shows an admixed microsatellite signature as displayed by Bayesian cluster analysis. Its genetic makeup looks like a mixture between Slovenian/SG and WG/NG individuals, which is supported by PCoA, suggesting that at least two introductions of mosquitoes into The Netherlands have taken place. However, the fact that the Dutch population shows a relatively large number of five *nad*4 haplotypes including one novel haplotype suggests a different conclusion: a large number of individuals with high genetic diversity could have been introduced recently from overseas. The genetic diversity may in this case be a measure of the time that has passed since the introduction. With time passing, founder populations normally experience a loss of genetic diversity, known as the founder effect [43]. If present, it should be possible to see this effect in future analyses of the Dutch *Ae. j. japonicus* population. Haplotypes H9 and H12, which account for 42% of the Dutch individuals, are the only haplotypes described by Fonseca et al. [27] for populations in Pennsylvania and Maryland.

Of the 18 Slovenian sampling localities examined, seven were located close to the Austrian border, eight close to the Croatian border and three in the centre of the country (cf. Figure 1). According to the Bayesian cluster analysis, all Slovenian/Croatian individuals show the same microsatellite signature of genotype 1 with only a little admixture of genotype 2 (Figure 2). Because the species is not yet widely distributed in Croatia, only two individuals were available. As with some specimens from other locations, these unfortunately could not be analysed due to their being heteroplasmic. Together with the Dutch samples, the Slovenian samples were the only ones in Europe to show *nad*4 haplotype H9. Additionally, some Slovenian individuals displayed haplotype H5, which is widely distributed in the NG and WG populations, indicating a possible link between these two and the Slovenian population.

In summary, the genetic information is not sufficient to decide whether the European *Ae. j. japonicus* have been introduced from the USA or from Japan, or from both countries. As the worldwide expansion of the species started almost two decades ago, exact source determination is becoming more and more difficult. Mitochondrial haplotypes are widespread, and in many cases it is impossible to say whether the species has found its way to Europe from Japan or from the USA.

In spite of this, all known *Ae. j. japonicus* populations in Europe must be assumed to have reached their infestation areas through human-mediated transport. Their apparent geographic separation does not permit any other conclusion. Individuals reach new regions passively and initially may manage to establish a relatively small population, depending on the number of founder individuals. When such populations merge, they can increase their genetic diversity and, accordingly, their adaptability. It is the mixed populations with high genetic diversity that are adaptable and likely to establish [44]. So far, six populations of *Ae. j. japonicus* have been found in Europe. Their genetic makeup shows a mixture of two genotypes, and most of the populations are expanding. The exception is the Belgian population, which seems to be very inbred and has a low genetic diversity [29]. At the same time, this population is the only one that has not expanded the area of infestation over the years since its introduction [45].

## Conclusions

Regarding intercontinental trade and travel as well as the transport of mosquitoes and pathogens between countries, it is to be expected that further populations of *Ae. j. japonicus* will appear in Europe and that the risk of pathogen transmission will increase. *Aedes j. japonicus* is known to feed on both birds and mammals, and bloodmeal analyses have shown that a high percentage (up to 60 %) of the identified blood sources were human [45]. Whilst *Ae. j. japonicus* has not attracted attention as an important vector in its native distribution area in East Asia, it was susceptible to several arboviruses in the laboratory, including WNV. A new study claims that German *Ae. j. japonicus* are refractory to WNV [46]. However, all the mosquitoes tested originated from a limited area in southern Germany, suggesting that a restricted and relatively homogeneous gene pool was included in the study. Other populations or subpopulations may very well be able to transmit WNV [19,47] or other pathogenic viruses.

There is consent that the eradication of *Ae. j. japonicus* from Europe is no longer possible, but efforts should be made to eliminate sources of introduction and to prevent the present populations from spreading further.

## Abbreviations

SG: South German(y)
NG: North German(y)
WG: West German(y)
WNV: West Nile virus
ECDC: European Centre for Disease Prevention and Control
PCR: Polymerase chain reaction
*nad*4: Sodium dehydrogenase subunit 4
PCoA: Principal coordinate analysis

## References

[1] Reinert JF, Harbach RE, Kitching IJ (2006). Phylogeny and classification of *Finlaya* and allied taxa (Diptera: Culicidae: Aedini) based on morphological data from all life stages. Zool J Linn Soc.

[2] GISD (Global invasive species database). http://www.issg.org/database/welcome, accessed 19 November 2014.

[3] Laird M, Calder L, Thornton R, Syme R, Holder P, Mogi M (1994). Japanese *Aedes albopictus* among four mosquito species reaching New Zealand in used tires. J Am Mosq Control Assoc.

[4] Peyton E, Campbell SR, Candeletti TM, Romanowski M, Crans WJ (1999). *Aedes* (*Finlaya*) *japonicus japonicus* (Theobald), a new introduction into the United States. J Am Mosq Control Assoc.

[5] Munstermann L, Andreadis T (1999). *Aedes japonicus* in Connecticut. Vector Ecol Newsl.

[6] Widdel AK, McCuiston LJ, Crans WJ, Kramer LD, Fonseca DM (2005). Finding needles in the haystack: single copy microsatellite loci for *Aedes japonicus* (Diptera: Culicidae). Am J Trop Med Hyg.

[7] Neitzel DF, Johnson KA, Brogren S, Kemperman MM (2009). First collection records of *Aedes japonicus* in Minnesota. J Am Mosq Control Assoc.

[8] Kampen H, Werner D (2014). Out of the bush: the Asian bush mosquito *Aedes japonicus japonicus* (Theobald, 1901)(Diptera: Culicidae) becomes invasive. Parasit Vectors.

[9] Thielman A, Hunter FF (2006). Establishment of *Ochlerotatus japonicus* (Diptera: Culicidae) in Ontario, Canada. J Med Entomol.

[10] Schaffner F, Chouin S, Guilloteau J (2003). First record of *Ochlerotatus* (*Finlaya*) *japonicus japonicus* (Theobald, 1901) in metropolitan France. J Am Mosq Control Assoc.

[11] Versteirt V, Schaffner F, Garros C, Dekoninck W, Coosemans M, Van Bortel W (2009). Introduction and establishment of the exotic mosquito species *Aedes japonicus japonicus* (Diptera: Culicidae) in Belgium. J Med Entomol.

[12] Schaffner F, Kaufmann C, Hegglin D, Mathis A (2009). The invasive mosquito *Aedes japonicus* in Central Europe. Med Vet Entomol.

[13] Becker N, Huber K, Pluskota B, Kaiser A (2011). *Ochlerotatus japonicus japonicus* – a newly established neozoan in Germany and a revised list of the German mosquito fauna. Eur Mosq Bull.

[14] Schneider K (2011). Breeding of *Ochlerotatus japonicus japonicus* (Diptera: Culicidae) 80 km north of its known range in southern Germany. Eur Mosq Bull.

[15] Seidel B, Duh D, Nowotny N, Allerberger F (2012). First record of the mosquitoes *Aedes* (*Ochlerotatus*) *japonicus japonicus* (Theobald, 1901) in Austria and Slovenia in 2011 and for *Aedes* (*Stegomyia*) *albopictus* (Skuse, 1895) in Austria 2012. Entomol Zeitschr.

[16] Kampen H, Zielke D, Werner D (2012). A new focus of *Aedes japonicus japonicus* (Theobald, 1901) (Diptera: Culicidae) distribution in Western Germany: rapid spread or a further introduction event?. Parasit Vectors.

[17] Werner D, Kampen H (2013). The further spread of *Aedes japonicus japonicus* (Diptera: Culicidae) towards northern Germany. Parasitol Res.

[18] Ibáñez-Justicia A, Kampen H, Braks M, Schaffner F, Steeghs M, Werner D (2014). First report of established population of *Aedes japonicus japonicus* (Theobald, 1901) (Diptera: Culicidae) in the Netherlands. J Eur Mosq Control Assoc.

[19] Sardelis MR, Turell MJ (2001). *Ochlerotatus j. japonicus* in Frederick County, Maryland: discovery, distribution, and vector competence for West Nile virus. J Am Mosq Control Assoc.

[20] Takashima I, Rosen L (1989). Horizontal and vertical transmission of Japanese encephalitis virus by *Aedes japonicus* (Diptera: Culicidae). J Med Entomol.

[21] Molaei G, Farajollahi A, Scott JJ, Gaugler R, Andreadis TG (2009). Human bloodfeeding by the recently introduced mosquito, *Aedes japonicus japonicus*, and public health implications. J Am Mosq Control Assoc.

[22] Sardelis MR, Dohm DJ, Pagac B, Andre RG, Turell MJ (2002). Experimental transmission of eastern equine encephalitis virus by *Ochlerotatus j. japonicus* (Diptera: Culicidae). J Med Entomol.

[23] Sardelis MR, Turell MJ, Andre RG (2002). Laboratory transmission of La Crosse virus by *Ochlerotatus j. japonicus* (Diptera: Culicidae). J Med Entomol.

[24] Turell MJ, Byrd BD, Harrison BA (2013). Potential for populations of *Aedes j. japonicus* to transmit Rift Valley fever virus in the USA. J Am Mosq Control Assoc.

[25] Schaffner F, Vazeille M, Kaufmann C, Failloux A-B, Mathis A (2011). Vector competence of *Aedes japonicus* for chikungunya and dengue viruses. Eur Mosq Bull.

[26] Brown WM, George M, Wilson AC (1979). Rapid evolution of animal mitochondrial DNA. Proc Natl Acad Sci U S A.

[27] Fonseca DM, Campbell S, Crans WJ, Mogi M, Miyagi I, Toma T (2001). *Aedes* (*Finlaya*) *japonicus* (Diptera: Culicidae), a newly recognized mosquito in the United States: analyses of genetic variation in the United States and putative source populations. J Med Entomol.

[28] ECDC (European Centre for Disease Prevention and Control) (2012). Guidelines for the surveillance of invasive mosquito species in Europe.

[29] Zielke DE, Werner D, Schaffner F, Kampen H, Fonseca DM (2014). Unexpected patterns of admixture in German populations of *Aedes japonicus japonicus* (Diptera: Culicidae) underscore the importance of human intervention. PLoS One.

[30] Schaffner F, Angel G, Geoffroy B, Hervy JP, Rhaiem A, Brunhes J (2001). *The mosquitoes of Europe* (CD-Rom).

[31] Gutsevich A, Monchadskii A, Shtakelberg A (1974). Fauna of the U.S.S.R. Diptera. Vol. 3, No. 4. Mosquitoes, family Culicidae.

[32] Fonseca DM, Widdel AK, Hutchinson M, Spichiger SE, Kramer LD (2010). Fine-scale spatial and temporal population genetics of *Aedes japonicus,* a new US mosquito, reveal multiple introductions. Mol Ecol.

[33] Egizi A, Fonseca D (2015). Ecological limits can obscure expansion history: patterns of genetic diversity in a temperate mosquito in Hawaii. Biol Invasions.

[34] Corpet F (1988). Multiple sequence alignment with hierarchical clustering. Nucleic Acids Res.

[35] Pritchard JK, Stephens M, Donnelly P (2000). Inference of population structure using multilocus genotype data. Genetics.

[36] Evanno G, Regnaut S, Goudet J (2005). Detecting the number of clusters of individuals using the software STRUCTURE: a simulation study. Mol Ecol.

[37] Earl DA, Vonholdt BM (2012). STRUCTURE HARVESTER: a website and program for visualizing STRUCTURE output and implementing the Evanno method. Conserv Genet.

[38] Peakall R, Smouse PE (2012). GenAlEx 6.5: genetic analysis in Excel. Population genetic software for teaching and research—an update. Bioinformatics.

[39] Sherwin WB (2010). Entropy and information approaches to genetic diversity and its expression: genomic geography. Entropy.

[40] Sherwin WB, Jabot F, Rush R, Rossetto M (2006). Measurement of biological information with applications from genes to landscapes. Mol Ecol.

[41] Dakin E, Avise J (2004). Microsatellite null alleles in parentage analysis. Heredity.

[42] Magnacca KN, Brown MJ (2010). Mitochondrial heteroplasmy and DNA barcoding in Hawaiian *Hylaeus* (*Nesoprosopis*) bees (Hymenoptera: Colletidae). BMC Evol Biol.

[43] Mayr E (1942). Systematics and the origin of species, from the viewpoint of a zoologist.

[44] Kolbe JJ, Glor RE, Schettino LR, Lara AC, Larson A, Losos JB (2004). Genetic variation increases during biological invasion by a Cuban lizard. Nature.

[45] Damiens D, Ayrinhac A, Van Bortel W, Versteirt V, Dekoninck W, Hance T (2014). Invasive process and repeated cross-sectional surveys of the mosquito *Aedes japonicus japonicus* establishment in Belgium. PLoS One.

[46] Huber K, Jansen S, Leggewie M, Badusche M, Schmidt-Chanasit J, Becker N (2014). *Aedes japonicus japonicus* (Diptera: Culicidae) from Germany have vector competence for Japan encephalitis virus but are refractory to infection with West Nile virus. Parasitol Res.

[47] Turell MJ, Sardelis MR, Dohm DJ, O'Guinn ML (2001). Potential North American vectors of West Nile virus. Ann N Y Acad Sci.

